# Carcinoma of the Lower Uterine Segment (LUS): Clinicopathological Characteristics and Association with Lynch Syndrome

**DOI:** 10.2174/138920211794520169

**Published:** 2011-03

**Authors:** Kenta Masuda, Kouji Banno, Megumi Yanokura, Yusuke Kobayashi, Iori Kisu, Arisa Ueki, Asuka Ono, Hiroyuki Nomura, Akira Hirasawa, Nobuyuki Susumu, Daisuke Aoki

**Affiliations:** Department of Obstetrics and Gynecology, Keio University School of Medicine, Tokyo, Japan

**Keywords:** Endometrial cancer, lower uterine segment, Lynch syndrome, DNA mismatch repair gene, *hMSH2.*

## Abstract

Endometrial cancer arises from the uterine body and fundus in many cases, but can also originate from the lower region of the uterine body through the upper region of the cervix. Such tumors are referred to as carcinoma of the lower uterine segment (LUS) or isthmus, and account for 3-6.3% of all cases of endometrial cancer. This relatively low incidence has permitted performance of only small-scale studies, but the clinical and pathological characteristics of carcinoma of the LUS in all these reports have differed from those of other endometrial cancers. Generally, endometrial cancer is classified into estrogen-dependent endometrioid adenocarcinoma (designated as type I), and non-endometrioid types that are less associated with estrogen and include poorly differentiated adenocarcinoma (type II). In some reports, carcinoma of the LUS has been found to have type II characteristics. Carcinoma of the LUS has also been associated with Lynch syndrome, a hereditary disease with frequent development of colorectal, endometrial, and ovarian cancers. Lynch syndrome is thought to be induced by mismatch repair gene mutation. The frequency of Lynch syndrome in cases of general endometrial cancer is 1-2%. In contrast, the frequency in patients with carcinoma of the LUS is much higher, with up to 29% of cases diagnosable with Lynch syndrome and a high frequency of *hMSH2* mutation found in one study. This suggests that further investigation of the clinical and pathological characteristics of carcinoma of the LUS and the association with Lynch syndrome is required through performance of a large-scale survey.

## INTRODUCTION

The incidence of endometrial cancer is highest among malignant tumors arising in the female genital organs. In the US, 40,000 patients are diagnosed with endometrial cancer annually, and 7,500 patients die of this disease [1]. The endometrium is pathologically divided into 2 regions: the uterine corpus proper (UC) and lower uterine segment (LUS) [2]. Endometrial cancer generally arises from the UC endometrium (the uterine body and fundus), but can originate from the LUS (the isthmus of the uterus) in rare cases. When a tumor localized in the LUS expands macroscopically from the lower uterine body through the upper cervix, it is regarded as carcinoma of the LUS. A tumor that is widely present from the uterine body through the endocervix is excluded from the definition of carcinoma of the LUS because the primary site cannot be determined with certainty [3]. Since the LUS is located between the uterine body and cervix, it shows histological characteristics of both the endometrium and uterine cervix in the glandular epithelium and interstitium. In addition, the LUS tends to respond poorly to hormone stimulation because the mucosal layer of the endometrium is thin compared to that of the uterine body [2].

Since endometrial cancer rarely develops from the LUS, only small-scale studies have been performed. However, the clinical and pathological characteristics of carcinoma of the LUS in these reports have differed from those of other endometrial cancers. Endometrial cancer is generally classified into 2 groups [4]: type I cases of estrogen-dependent endometrioid adenocarcinoma, which account for 70-80% of endometrial cancer cases; and type II cases, including non-endometrioid types and poorly differentiated adenocarcinoma that are less associated with estrogen, which account for 10-20% of endometrial cancer cases. Continuous estrogen stimulation (unopposed estrogen) of the endometrium in the absence of progesterone is considered to be the cause of type I tumors, whereas type II cases arise from an atrophied endometrium and are often unrelated to estrogen. In some reports, carcinoma of the LUS lacks type I characteristics, but tends to show type II characteristics [5], which is assumed to be due to the thin endometrial layer of the LUS and a weak endometrial response to estrogen. Clinically, type I cases are characterized by irregular menstruation inducing unopposed estrogen, nulliparity, infertility, and a high frequency of PCOS (polycystic ovary syndrome). In contrast, type II cases show weak expression of estrogen and progesterone receptors, and p53 abnormality, and carcinoma of the LUS shows similar characteristics [3, 6-9].

The association of carcinoma of the LUS with a hereditary tumor, Lynch syndrome, has attracted recent attention. Lynch syndrome is a hereditary disease in which there is frequent development of colorectal, endometrial, and ovarian cancers. The cause is thought to be a mismatch repair (MMR) gene mutation in germ cells and the frequency in cases of general endometrial cancer is 1-2% [10]. In contrast, Lynch syndrome has a high frequency in cases with carcinoma of the LUS, with one report in the US suggesting that 29% of cases could also be diagnosed with Lynch syndrome and that the *hMSH2* mutation was present at a high frequency [11]. Demonstration of an association between carcinoma of the LUS and Lynch syndrome in a large-scale survey would allow patients with carcinoma of the LUS to be classified as a high-risk group for Lynch syndrome.

## PATHOLOGICAL DEFINITION AND CHARACTERISTICS OF CARCINOMA OF THE LUS

A tumor localized in the LUS that expands macroscopically from the lower uterine body through the upper cervix is regarded as carcinoma of the LUS. A cancer that is widely present from the body through the endocervix is excluded from the definition of carcinoma of the LUS because the primary site cannot be identified [3]. The cavity of the uterus is histologically divided into 2 regions: the mucosa of the LUS, and that of the uterine body. The endometrium of the LUS is generally thinner than that of the fundus, and glands and interstitium in this region tend to respond slowly to hormone stimulation, which is thought to delay endometrial development. There are 2 types of cells in the superficial and glandular epithelia in the isthmus of the uterus: columnar and ciliated cells. The endometrial layer of the LUS is similar to the endometrium with regard to cell distribution and histochemistry, but the volume of the endometrium tends to be smaller in the LUS [12]. Since the endocervical mucosa gradually transits to the endometrium of the LUS histologically, endocervical and endometrial features are mixed in the glandular epithelium and interstitium in the LUS [2].

Pathologically, 80% of cases of endometrial cancer are classified as endometrioid adenocarcinoma. Histological grading for differentiation is applied only for endometrioid adenocarcinoma, and lesions are classified into G1 to G3 based on the proportion of the solid region (FIGO staging). Squamous differentiation is a common finding. Such type I cases of endometrioid adenocarcinoma are moderately to well differentiated and often arise from background endometrial hyperplasia due to long-term excess estrogen stimulation. About 10% of cases of endometrial cancer are classified as type II and have a higher risk of recurrence and metastasis compared to type I cases. Type II cases are not related to estrogen, but are caused by an atrophied endometrium [4].

## DISCRIMINATION BETWEEN CARCINOMA OF THE LUS AND CERVICAL ADENOCARCINOMA

It is often difficult to determine whether widely expanded adenocarcinoma centered in the uterine isthmus originated from the uterine body or cervix, even after pathological evaluation of a biopsied specimen. However, identification of the origin is important to establish an appropriate treatment policy. After diagnosis of endometrial cancer, surgery is performed, the stage is identified, and adjuvant therapy is administered. In contrast, when cervical cancer is diagnosed, the clinical stage is identified before surgery, and chemoradiotherapy without surgery is recommended for stages Ib, II or higher [13].

There have been several reports on discrimination of endometrial cancer from cervical cancer. Using MRI, Haider *et al*. found that endometrial hypertrophy, endometrial tumor, tumor advancement in the uterus, and muscular invasion *via *the endometrium occurred at a significantly higher frequency in endometrial cancer than in cervical adenocarcinoma, and that these characteristics were useful for discriminating between the two conditions [14]. In contrast, Westin *et al*. found that MRI was not necessarily useful for preoperative discrimination [11]. Immunohistologically, typical cases of endometrial cancer are positive for ER and vimentin and negative for CEA, whereas cervical cancer shows the opposite pattern of negative for ER and vimentin and positive for CEA. Various small-scale studies have examined the optimum combination of markers [15].

Detection of HPV DNA and immunostaining for p16 may also be useful for discriminating cervical adenocarcinoma from endometrial cancer. HPV DNA is detected in cervical adenocarcinoma at a higher frequency than in endometrial cancer. The *p16* gene codes for a cyclin-dependent kinase inhibitor and there is a high risk of HPV infection with *p16* overexpression in cervical cancer. Expression of p16 protein also occurs in endometrial cancer based on immunostaining and may be associated with prognosis. Comparison of immunostaining results indicated diffuse *p16* overexpression in cervical cancer, but spotted and weak expression in endometrial cancer, and this may allow discrimination between the two diseases [16].

## CLINICAL CHARACTERISTICS OF CARCINOMA OF THE LUS

The important prognostic factors in endometrial cancer are histologic type, grade of differentiation, and FIGO surgical stage [17]. Investigation of the pathological characteristics of carcinoma of the LUS in comparison with non-LUS endometrial cancer is important to understand its characteristics and prognosis. In 12 Japanese patients with carcinoma of the LUS, Hachisuga *et al*. [18] found a histologic type of adenosquamous carcinoma in 7 (58%) and differentiation to grade 3 in 7 (58%), both of which were higher than the respective frequencies of 6% and 12% in non-LUS endometrial cancer. Muscular invasion was found in all 12 patients [18]. In 5 patients with carcinoma of the LUS, Jacques *et al*. found that all were grade 3, with a histologic type of endometrioid adenocarcinoma accompanied by differentiation to squamous epithelium in 3, a mixed tumor of serous and clear cell adenocarcinomas in 1, and MMMT in 1. All 5 patients died within 23 months after diagnosis [3].

In 13 patients with carcinoma of the LUS, Watanabe *et al*. found no significant differences in the frequencies of histologic types or differentiation grades, but higher rates of invasion of half or more of the muscular layer, lymph node metastasis, and positive ascites in cytology compared to non-LUS cases [6]. Westin *et al*. also found no significant differences in the frequencies of concomitant endometrial hyperplasia, histologic types, or grades in 35 patients with carcinoma of the LUS, but a higher frequency of stage II, deeper muscular invasion, a higher frequency of lymphovascular space invasion, and a smaller tumor diameter compared to non-LUS cases [11]. In a study limited to patients with endometrial cancer who were younger than 50 years old, the frequency of endometrioid adenocarcinoma differentiation to squamous epithelium was higher, muscular invasion was deeper, the grade was higher, and the frequency of concomitant endometrial hyperplasia was lower in 16 patients with carcinoma of the LUS compared to cases of non-LUS endometrial cancer, but there was no difference in the 10-year survival rate [5].

The frequency of carcinoma of the LUS has been found to be 3-6.3% of all endometrial cancer cases [3, 11, 18]. In the largest comparison study (35 patients with carcinoma of the LUS vs. 974 patients with non-LUS endometrial cancer), the mean onset ages were 54.2 and 62.9 years old, respectively, with a significantly younger age of onset for carcinoma of the LUS [11]. The age of onset has varied in other studies [3, 5], but the proportion of cases of carcinoma of the LUS increased to 18% (16/88) in patients limited to those younger than 50 years old [5].

Regarding risk factors, the cause of type I endometrial cancer is unopposed estrogen. Obesity increases insulin resistance and an elevation of the blood estradiol level promotes endometrial growth, leading to a risk of endometrial cancer that is 2 and 3 times greater with a BMI higher than 25 and 30, respectively [19]. Nulliparity, amenorrhea, and infertility cause long-term estrogen stimulation and are risk factors for endometrial cancer. In a comparison of 16 patients with carcinoma of the LUS and 72 patients with non-LUS endometrial cancer, Hachisuga *et al*. found that the frequencies of irregular menstruation, nulliparity, infertility, and PCOS were significantly lower in those with carcinoma of the LUS, showing that carcinoma of the LUS lacks the characteristics of typical type I endometrial cancer [5].

The influence on prognosis of endometrial cancer dissemination to the LUS has also been investigated. Since stage II endometrial cancer is accompanied by cervical invasion and expands to the LUS in most cases, most studies have been limited to patients with stage I endometrial cancer and have compared the prognosis between cases with (rates of 28-58%) and without LUS involvement [20-23]. Phelan *et al*. found that grade, lymphovascular space invasion, muscular invasion, and histologic type, but not LUS involvement, were related to the recurrence rate in stage I endometrial cancer [20]. Brown *et al*. also found that LUS involvement was not related to progression-free survival or recurrence rate [21], but that cases with LUS involvement had a high rate of lymph node metastasis [23].

## CARCINOMA OF THE LUS AND HORMONE RECEPTORS

Expression levels of estrogen (ER) and progesterone (PR) receptors change with the menstrual cycle in the normal endometrium, as shown by immunohistological staining. ER and PR are also expressed in type I endometrial cancer, which accounts for most cases of sporadic endometrioid adenocarcinoma and is associated with unopposed estrogen activity. In contrast, no or weak expression of ER and PR occurs in many cases of type II endometrial cancer [7]. Many studies have shown an association of ER and PR expression with the prognosis of endometrial cancer. However, some have shown that ER and PR levels are independent prognostic factors, whereas others found a poor association with the prognosis; thus, the prognostic utility of ER and PR in endometrial cancer is unclear [24]. In carcinoma of the LUS, Watanabe *et al*. found ER- and PR-positive rates of 23.1% (3/13) and 7.7% (1/13), respectively, compared to rates of 54.5% (18/33) for both receptors in non-LUS endometrial cancer, showing a significant reduction of expression in carcinoma of the LUS [6]. Jacques *et al*. also found ER- and PR-positive rates of only 20% (1/5) in carcinoma of the LUS [3]. In contrast, Westin *et al*. reported an ER-positive rate of 92% (24/26) in carcinoma of the LUS, with 73% (19/26) of the cases found to be ER-positive, vimentin-positive, and CEA-negative, similarly to the typical findings for endometrioid adenocarcinoma of the uterine body [11]. These results indicate that a consistent conclusion has not been reached. 

## CARCINOMA OF THE LUS AND p53

*p53* is a tumor suppressor gene located on chromosome 17. p53 protein inhibits proliferation and induces apoptosis of cells with DNA damage. Abnormal p53 is thought to be involved in carcinogenesis of endometrial cancer, and particularly in type II endometrial cancer including serous adenocarcinoma. A *p53* mutation has been observed in more than 60% of cases immunopositive for p53, and immunohistochemical detection of p53 serves as a prognostic factor for endometrial cancer since it indicates a functional p53 abnormality [25]. Abnormal p53 expression was more frequently observed in non-estrogen dependent type II endometrial cancer and poorly differentiated (Grade 3) type I endometrial cancer [8]. In carcinoma of the LUS, Jacques *et al*. found that 60% (3/5) of cases had abnormal p53 protein [3]; Jiko *et al*. found a p53 mutation in 38% (3/8) of cases [9]; and Watanabe *et al*. found overexpression of p53 in 61.5% (8/13) of cases, a frequency higher than that (18.2%, 6/33) in non-LUS endometrial cancer [6]. p53 mutations also occur in cervical adenocarcinoma, but Jiko *et al*. stated that the p53 point mutation pattern in carcinoma of the LUS is more similar to that in endometrial cancer than in cervical adenocarcinoma [9].

## CARCINOMA OF THE LUS AND LYNCH SYNDROME

Lynch syndrome or HNPCC is a hereditary disease that includes frequent development of colorectal, endometrial, and ovarian cancers. Lynch syndrome is caused by a hereditary defect in the mismatch repair (MMR) gene and the incidences in colorectal and endometrial cancers are 2-3% and 1.8-2.1%, respectively [10]. In Lynch syndrome with a *hMLH1* or *hMSH2* mutation, the frequencies of colorectal and endometrial cancers are 68 and 62%, respectively, and the lifetime risk of developing endometrial cancer is higher than that for colorectal cancer in women [26]. In patients with Lynch syndrome, there have been fewer studies on endometrial cancer compared to colorectal cancer due to problems with screening. The 1999 revised Amsterdam criteria II include endometrial cancer as a Lynch syndrome-related tumor, but women who develop endometrial cancer as the initial cancer and patients with a family tree with insufficient detail are not included, and a high false negative rate has been reported based on these criteria [26]. For colorectal cancer, the Bethesda criteria require microsatellite instability (MSI) testing, but this is not applicable for patients who develop endometrial cancer as the initial cancer. Thus, there is a need to establish criteria for selection of patients with endometrial cancer who should undergo screening [27].

Westin *et al*. [11] diagnosed Lynch syndrome meeting the Amsterdam criteria II in 14.2% (5/35) of cases with carcinoma of the LUS. A *hMSH2 *gene mutation was present in all 5 cases, with MSI and a reduced hMSH2 protein level on immunostaining. Four further cases showed reduced hMSH2 and hMSH6 levels on immunostaining and high MSI, strongly suggesting *hMSH2 *gene mutation, but did not meet the Amsterdam criteria II; and one case showed a reduced hMLH1 level despite the absence of aberrant DNA hypermethylation. All 10 patients (29%) were regarded to have Lynch syndrome [11]. The frequency of Lynch syndrome in carcinoma of the LUS was 14.2% even if limited to the 5 cases with definite Lynch syndrome, which is very high compared to the frequency of 1-2% in general endometrial cancer.

In an immunohistological study of expression of mismatch repair gene-encoded proteins (hMLH1, hMSH2, hMSH6, and PMS2), Garg *et al*. found carcinoma of the LUS in 5 of 32 patients with reduced protein expression, but only in 1 of 39 with normal protein expression, indicating an association between carcinoma of the LUS and Lynch syndrome [28]. On the other hand, Watanabe *et al*. found that 24.2% (8/33) of non-LUS endometrial cancer cases were MSI-H compared to 0% (0/13) of carcinoma of the LUS cases in MSI analysis [6], suggesting a possible difference in genetic background between US and Japanese patients [11]. Only Westin *et al*. have previously proposed an association between carcinoma of the LUS and Lynch syndrome, and a further investigation of this association is required. 

## CONCLUSION

Only small-scale studies on carcinoma of the LUS have been reported due to the rarity of this tumor among endometrial cancers. Our review of these reports suggested that carcinoma of the LUS may show the clinicopathological characteristics of type II endometrial cancer and that the frequency of associated Lynch syndrome may be high. Verification of these findings is required through comparison of carcinoma of the LUS and general endometrial cancer in a large-scale study to investigate the dependence of the histologic type and prognosis on the developmental site of endometrial cancer, and to determine the frequency of Lynch syndrome in carcinoma of the LUS. A finding of a high frequency will allow carcinoma of the LUS to be defined as a risk factor for Lynch syndrome, which may increase the screening sensitivity for Lynch syndrome.
